# Association Between Irinotecan-Containing Regimens and Functional and Nutritional Impairment in Advanced Colorectal Cancer: An Exploratory PG-SGA Analysis

**DOI:** 10.3390/cancers18071140

**Published:** 2026-04-01

**Authors:** Jorge Guerrero-Martín, Raquel Macias-Montero, Yolanda Macías-Gañán, Marta Araujo-Blesa, María Sandra Paniagua-Vivas, Luis Enrique Sánchez-Diestro

**Affiliations:** 1 Faculty of Medicine and Health Sciences, University of Extremadura, 06071 Badajoz, Spain; jorguerr@unex.es; 2 Spain Coordinator of the Biomedical and Sociosanitary Research, Teaching and Innovation Group (GIDIBS), Faculty of Medicine, University of Extremadura, 06071 Badajoz, Spain; 3 Department of Biomedical Sciences, Faculty of Medicine and Health Sciences, University of Extremadura, 06071 Badajoz, Spain; raquel.macias@salud-juntaex.es; 4 Department of Medical Oncology, University Hospital Complex of Badajoz, Extremadura Health Service, 06080 Badajoz, Spain; 5 Department of Medical-Surgical Therapeutics, Pharmacology Area, University Center of Plasencia, 10600 Plasencia, Spain; yolandamg@unex.es; 6 Research Group PAIDI-CTS-1141:“Applied Clinical Research in Care and New Healthcare Paradigms (ICCAPA)”, Department of Nursing, Faculty of Nursing, Physiotherapy and Podiatry, University of Seville, 41009 Seville, Spain; maraujo1@us.es; 7 Department of Nursing, University Centre of Mérida, University of Extremadura, 06800 Mérida, Spain; mariasanpv@unex.es

**Keywords:** colorectal cancer, irinotecan, malnutrition, patient-generated subjective global assessment (PG-SGA), functional status

## Abstract

Background/Objectives: Malnutrition is highly prevalent in patients with advanced colorectal cancer (CRC) and is associated with poorer treatment tolerance, reduced quality of life, and worse clinical outcomes. Chemotherapy regimens incorporating irinotecan are commonly used in later treatment lines but present a distinct toxicity profile characterized mainly by gastrointestinal symptoms and functional decline, which may negatively affect nutritional status. This study aimed to evaluate the impact of irinotecan-containing chemotherapy regimens on nutritional status and functional capacity in patients with advanced CRC using the Patient-Generated Subjective Global Assessment (PG-SGA). Methods: A cross-sectional observational study was conducted in 91 adult patients with histologically confirmed stage III–IV colorectal adenocarcinoma undergoing active systemic chemotherapy between November 2023 and June 2024. Demographic and clinical variables, treatment regimens, and exposure to irinotecan were recorded. Nutritional status was assessed using the PG-SGA, and body composition was evaluated by multifrequency bioelectrical impedance analysis. Associations between irinotecan exposure and nutritional, functional, and symptom-related variables were analyzed using χ^2^ or Fisher’s exact tests. Results: Thirteen patients (14.3%) received irinotecan-containing regimens, most frequently combined with FOLFOX. Patients exposed to irinotecan presented higher PG-SGA scores, indicating a greater nutritional burden. Although most gastrointestinal symptoms did not reach statistical significance, early satiety (*p* = 0.041) and functional deterioration (*p* = 0.039) were significantly associated with irinotecan administration, while nausea (*p* = 0.089) and vomiting (*p* = 0.087) showed trends toward significance. The subgroup treated with FOLFOX–irinotecan also demonstrated a higher frequency of functional impairment compared with those receiving FOLFOX alone. Conclusions: Irinotecan-containing chemotherapy regimens in advanced CRC may be associated with a distinct pattern of nutritional deterioration primarily driven by functional decline and gastrointestinal symptoms affecting food intake. Systematic nutritional assessment using validated tools such as the PG-SGA may allow early identification of vulnerable patients and support the implementation of timely multimodal interventions aimed at improving treatment tolerance and clinical outcomes.

## Simple Summary

Many patients with advanced colorectal cancer experience poor nutrition, which can make treatments harder to tolerate and worsen overall health and quality of life. Some chemotherapy options used in later stages, particularly those including irinotecan, are known to cause side effects such as digestive problems and reduced physical functioning, which may further affect a patient’s ability to eat and stay well nourished. This study explores how these treatments influence nutritional status and daily functioning in people receiving chemotherapy for advanced colorectal cancer. By using a standardized assessment tool, the researchers aim to better identify patients at risk of nutritional decline. The findings suggest that certain treatment combinations may be linked to worsening appetite, early fullness, and reduced physical capacity. Understanding these effects can help healthcare professionals detect problems earlier and provide timely nutritional and supportive care, ultimately improving patients’ ability to cope with treatment and enhancing clinical outcomes.

## 1. Introduction

Colorectal cancer (CRC) continues to represent one of the leading causes of cancer-related morbidity and mortality worldwide. The increasing prevalence of the disease, together with the growing number of therapies used throughout the course of the neoplastic process, has led to a greater demand for healthcare resources [1,2]. In this context, in advanced/metastatic colorectal cancer, systemic therapy is commonly based on fluoropyrimidine-, oxaliplatin- and irinotecan-containing regimens, often combined with biologic or molecularly targeted agents according to the tumor molecular profile and treatment goals. In contrast, treatment strategies in adjuvant and neoadjuvant settings are more disease-stage specific and should be described separately [3].

Among the available regimens, FOLFOX and XELOX have been widely used as first-line treatments, while irinotecan—an inhibitor of topoisomerase I—plays a key role in later treatment lines or as a therapeutic alternative in regimens such as FOLFIRI, particularly in patients who progress after exposure to oxaliplatin [4] or when oxaliplatin is contraindicated. However, the toxicity profile of irinotecan differs substantially from that of oxaliplatin-based regimens, with a predominance of gastrointestinal and systemic adverse effects, particularly severe diarrhea, anorexia, asthenia, and persistent fatigue [5,6].

Beyond their immediate impact on treatment tolerance, these adverse effects have clinically relevant consequences for the patient’s nutritional status and functional capacity. Irinotecan-associated gastrointestinal toxicity may contribute to reduced intake, greater symptom burden and functional decline, which in turn may be associated with poorer nutritional status and clinical vulnerability [7,8]. This impact is especially significant in patients previously treated with FOLFOX or XELOX, in whom cumulative toxicity may further increase the risk of malnutrition and clinical frailty [9].

Cancer-related malnutrition is an independent prognostic factor associated with increased toxicity, a higher rate of complications, reduced overall survival, and significant deterioration in quality of life [10,11]. In patients with CRC undergoing chemotherapy, malnutrition prevalence may exceed 60–70%, particularly in those with advanced disease, persistent gastrointestinal symptoms, or progressive functional decline. These estimates are primarily derived from studies of selected symptomatic cohorts and specific nutritional assessment frameworks [12]. Despite this high incidence, systematic nutritional assessment is still not fully integrated into many oncological care protocols.

In this context, the Patient-Generated Subjective Global Assessment (PG-SGA) is one of the most widely validated and recommended oncology-specific nutritional assessment tools, as it allows a comprehensive assessment including weight loss, changes in dietary intake, nutrition-impact symptoms, and—particularly relevant—functional capacity [13]. This functional dimension plays a central role in patients treated with irinotecan, as it reflects not only nutritional status but also the interaction between pharmacological toxicity, systemic inflammation, and loss of muscle mass.

In a previous study, Sánchez-Diestro et al. systematically evaluated nutritional status and symptoms associated with nutritional risk in patients with colorectal cancer treated mainly with oxaliplatin-based regimens such as FOLFOX and XELOX, using the PG-SGA as the central screening and assessment tool [14]. That study identified a high prevalence of malnutrition and characterized differential toxicity profiles associated with these regimens. However, although irinotecan was present in a limited proportion of the cohort, its specific analysis was not the primary objective of the study, which limits the extrapolation of the results to this drug.

Irinotecan presents a distinctive toxicity profile characterized by a high burden of gastrointestinal symptoms and a potentially greater impact on functional capacity, particularly when administered in later lines of treatment. Evidence specifically focused on its nutritional and functional repercussions remains scarce, and few studies have addressed this impact from a comprehensive perspective using validated tools such as the PG-SGA.

Because irinotecan-containing regimens have a toxicity profile distinct from oxaliplatin-based regimens, particularly regarding gastrointestinal symptoms and functional burden, it remains unclear whether they may be associated with a different nutritional deterioration phenotype when assessed using a structured tool such as PG-SGA. The present study therefore explored the association between irinotecan exposure and nutritional/functional impairment in patients with advanced colorectal cancer.

## 2. Materials and Methods

### 2.1. Study Population and Data Sources

An analytical observational cross-sectional study was conducted in a cohort of adult patients with a histopathologically confirmed diagnosis of colorectal adenocarcinoma in locally advanced or metastatic stages (stage III–IV) who were receiving active systemic chemotherapy regimens that included irinotecan as one of their components.

The study was carried out in the Medical Oncology Department of the University Hospital of Badajoz (Spain) within the framework of routine clinical practice between November 2023 and June 2024. Patients aged ≥18 years with sufficient cognitive capacity to participate in the structured clinical interview and to complete the nutritional assessment instruments were included, provided that they gave written informed consent.

Patients with severe concomitant medical conditions unrelated to the oncological process that could significantly alter baseline nutritional status or functional capacity were excluded. These included advanced organ failure, active systemic inflammatory diseases, or severe infectious processes.

Data were obtained through a directed clinical interview, systematic review of the electronic medical record, and nutritional and body composition assessment using validated tools.

### 2.2. Baseline Demographic and Clinical Variables

Baseline demographic and clinical variables were recorded, including age, sex, body mass index (BMI), location of the primary tumor (colon or rectum), and tumor stage, categorized as stage III or IV according to the current TNM classification.

Age was analyzed both as a continuous and categorical variable due to its clinical relevance as a factor influencing irinotecan-related toxicity, functional reserve, and nutritional vulnerability.

### 2.3. Variables Related to Therapeutic Exposure

A detailed characterization of the systemic oncological treatments administered was performed, with particular emphasis on the inclusion of irinotecan within specific therapeutic regimens and its potential contribution to the overall toxicity profile of treatment.

The complete therapeutic sequence was documented, including prior or subsequent exposure to oxaliplatin-based regimens (FOLFOX or XELOX), in order to explore whether the administration of irinotecan within a given treatment sequence could be associated with nutritional and functional deterioration that might lead to poorer clinical tolerance to FOLFOX, as indirectly suggested in the previous study.

Therapeutic intent (adjuvant, neoadjuvant, or palliative) and the concomitant use of targeted therapies (bevacizumab, cetuximab, or panitumumab), when clinically indicated, were also recorded.

From a clinical standpoint, the occurrence of adverse events typically associated with irinotecan was systematically evaluated, including early and late diarrhea, anorexia, nausea, vomiting, mucositis, abdominal pain, marked asthenia, persistent fatigue, and deterioration of functional capacity. These effects were considered potential mediators of the negative impact of irinotecan administration on nutritional status and overall treatment tolerability.

### 2.4. Nutritional Status and Body Composition Assessment

Nutritional status was assessed using the Patient-Generated Subjective Global Assessment (PG-SGA), a validated tool in oncological populations that integrates information on weight loss, changes in dietary intake, nutrition-impact symptoms, and evaluation of functional capacity, a component of particular interest in patients exposed to irinotecan.

The total score was interpreted according to internationally accepted cut-off points, determining the level of nutritional intervention required.

Additionally, body composition analysis was performed using multifrequency segmental bioelectrical impedance analysis with the TANITA^®^ RD-545 device (TANITA Europe). This equipment estimates body composition based on the measurement of impedance generated by the passage of a low-intensity alternating current, allowing the assessment of parameters such as fat-free mass, total fat mass, skeletal muscle mass, bone mass, and basal metabolic rate, with segmental analysis of the trunk and extremities.

Results were interpreted according to reference values adjusted for age and sex, as well as consensus clinical cut-off points for the identification of muscle mass loss and risk of malnutrition.

### 2.5. Statistical Analysis

Statistical analysis was performed using JASP software (version 0.17.0.0), based on the R environment. Continuous variables are described using mean and standard deviation or median and interquartile range depending on data distribution, which was assessed using the Kolmogorov–Smirnov test.

Categorical variables are expressed as absolute frequencies and percentages. To analyze the association between the inclusion of irinotecan in the therapeutic regimen and nutritional, functional, and body composition parameters, χ^2^ tests or Fisher’s exact test was used. Categorical variables are expressed as absolute frequencies and percentages. Associations between irinotecan exposure and categorical variables were assessed using the χ^2^ test when expected cell counts were adequate. When the expected frequency in any cell was <5, Fisher’s exact test was applied to account for sparse data conditions.

Given the limited number of patients exposed to irinotecan (*n* = 13), sparse contingency tables were anticipated in several comparisons; therefore, Fisher’s exact test was preferentially used in these cases to ensure validity of the results.

Additionally, the relationship between exposure to irinotecan and clinical and functional tolerance to oxaliplatin-based regimens (FOLFOX) was explored through comparative analyses of nutritional and functional indicators. A *p*-value < 0.05 was considered statistically significant.

### 2.6. Ethical Considerations

The study was conducted in accordance with the ethical principles of the Declaration of Helsinki and current biomedical research legislation. All participants received detailed information about the objectives and procedures of the study and signed informed consent prior to inclusion.

The protocol was approved by the Ethics Committee of the Badajoz Health Area Management. Data were anonymized and treated confidentially, and were used exclusively for scientific purposes.

## 3. Results

### 3.1. Demographic and Clinical Characteristics of the Cohort

A total of 91 patients with a diagnosis of colorectal adenocarcinoma receiving active chemotherapy treatment were included. No losses or refusals were recorded in the completion of the Patient-Generated Subjective Global Assessment (PG-SGA); therefore, all subjects were included in the final analysis.

Of the total sample, 35 patients (38.46%) were women and 56 (61.54%) were men.

**Figure 1:**
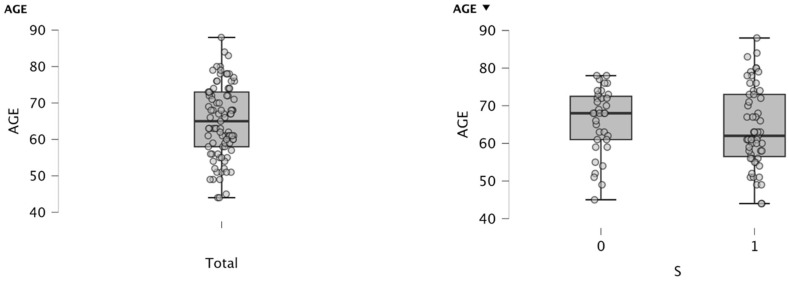
Box plot (median, Q1–Q3, min–max) showing the age distribution of the patients included in the study sample and box plot of age categorized by sex in the study sample. Source: Own elaboration.

The overall mean age was 64.97 ± 9.92 years, with an interquartile range between 58 years (P25) and 73 years (P75) (Figure 1).

When stratified by sex, the mean age in men was 64.27 ± 10.69 years, while in women it was 66.00 ± 8.77 years. No clinically relevant differences were observed in the age distribution between the two groups (Figure 1).

### 3.2. Distribution of Therapeutic Regimens

Regarding the treatment received, 42 patients (46.2%) were undergoing treatment with XELOX and 36 patients (39.6%) with FOLFOX. Thirteen patients (14.3%) were receiving irinotecan, administered in combination with FOLFOX in 10 cases (76.9% of those treated with irinotecan) and in combination with XELOX in 3 cases (23.1%).

Therefore, the subgroup treated exclusively with FOLFOX consisted of 26 patients, while the FOLFOX–irinotecan subgroup included 10 patients. All patients were evaluated using multifrequency bioelectrical impedance analysis and the PG-SGA.

### 3.3. PG-SGA Results According to Irinotecan Exposure

Analysis of the total PG-SGA score showed a greater unfavorable nutritional burden in patients treated with irinotecan, particularly in those receiving it in combination with FOLFOX.

**Figure 2:**
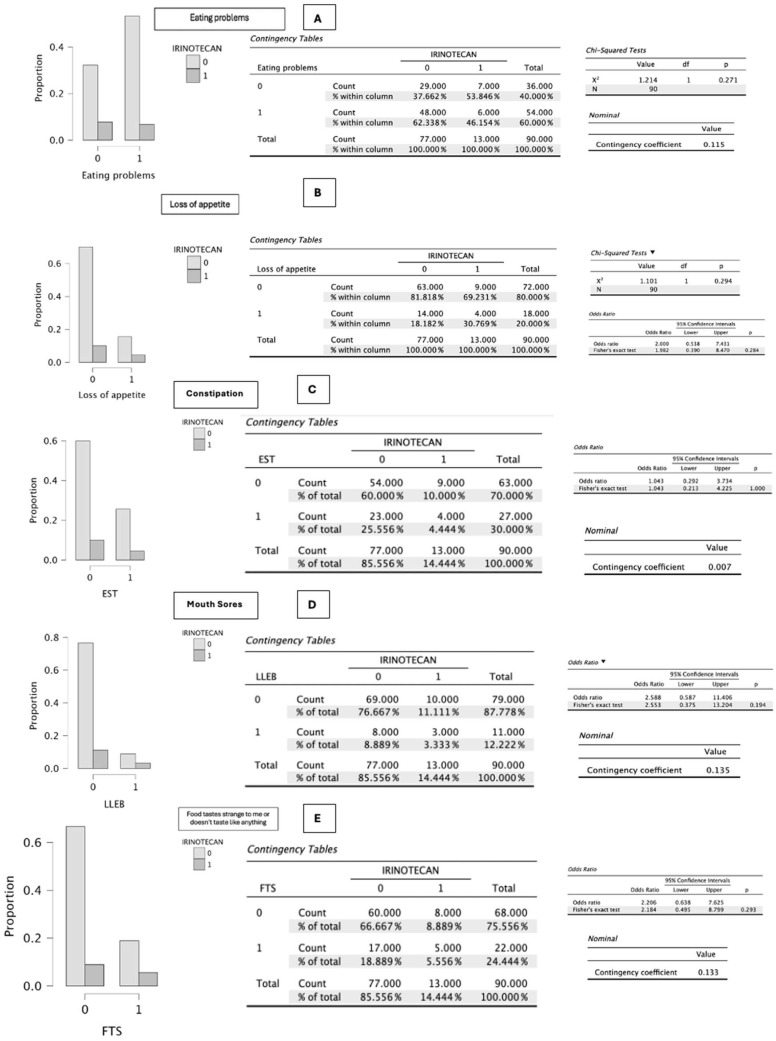
(**A**–**E**) Graphical representation of the difference in proportions between patients who present eating problems, loss of appetite, constipation, oral sores, and taste alterations (foods taste unusual or have no taste) and who receive irinotecan—regardless of the treatment with which it is combined—compared with those who do not receive it. A value of 0 indicates no irinotecan administration, and 1 indicates irinotecan administration. The *p*-value obtained according to the χ^2^ test or Fisher’s exact test is shown. *p*-values were obtained using χ^2^ or Fisher’s exact test as appropriate according to cell distribution. Source: Own elaboration.

**Figure 3:**
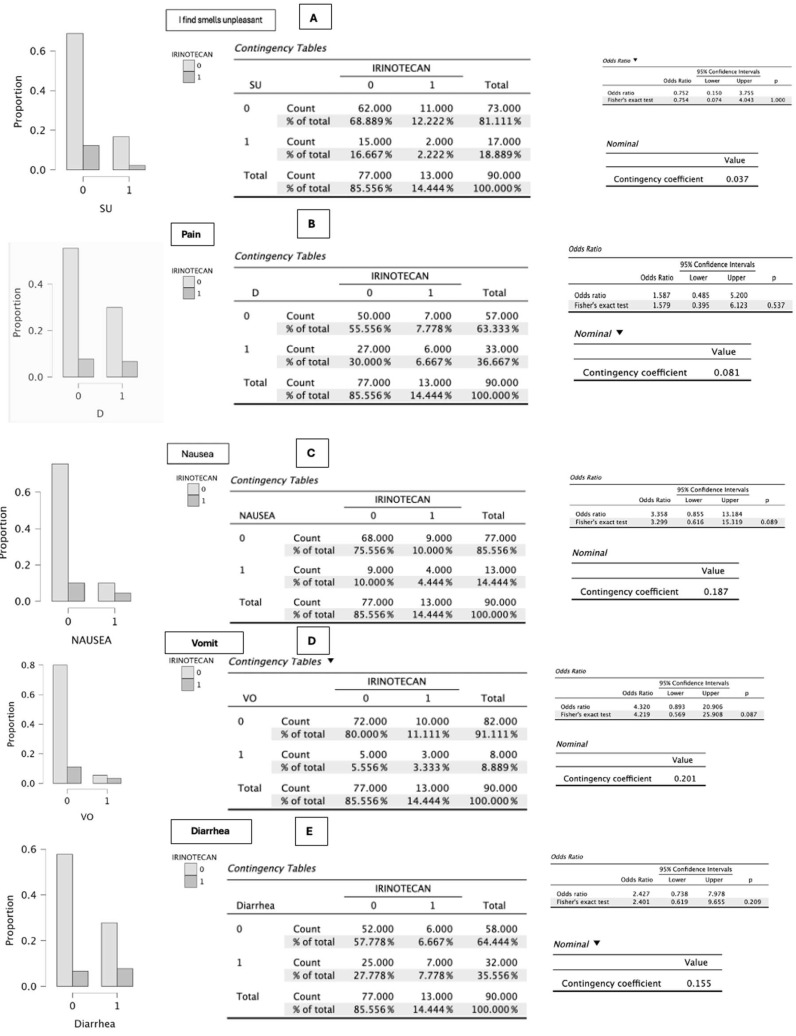
(**A**–**E**) Graphical representation of the difference in proportions between patients who report unpleasant odors, pain, nausea, vomiting, and diarrhea and who receive irinotecan—regardless of the treatment with which it is combined—compared with those who do not receive it. A value of 0 indicates no irinotecan administration, and 1 indicates irinotecan administration. The *p*-value obtained according to the χ^2^ test or Fisher’s exact test is shown. *p*-values were obtained using χ^2^ or Fisher’s exact test as appropriate according to cell distribution. Source: Own elaboration.

**Figure 4:**
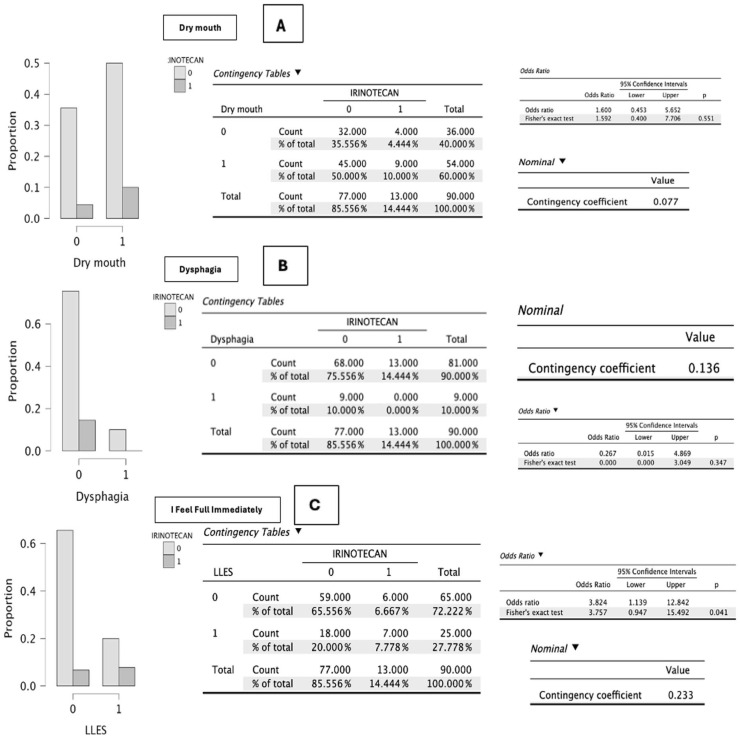
(**A**–**C**). Graphical representation of the difference in proportions between patients who report dry mouth, dysphagia, and early satiety during meals and who receive irinotecan—regardless of the treatment with which it is combined—compared with those who do not receive it. A value of 0 indicates no irinotecan administration, and 1 indicates irinotecan administration. The *p*-value obtained according to the χ^2^ test or Fisher’s exact test is shown. *p*-values were obtained using χ^2^ or Fisher’s exact test as appropriate according to cell distribution. Source: Own elaboration.

**Figure 5:**
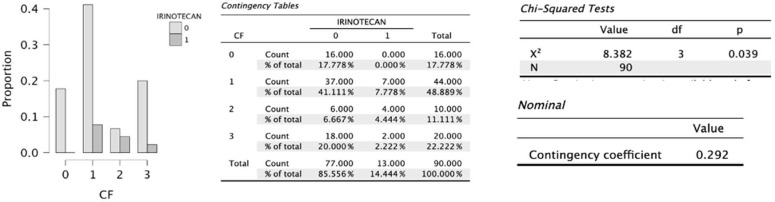
Graphical representation of the significant difference in proportions across the different levels of functional capacity (FC) among patients in the study sample who receive irinotecan compared with those who do not, where 0 indicates no irinotecan administration and 1 indicates irinotecan administration. The *p*-value was obtained according to the χ^2^ test. Functional capacity levels are defined as follows: 0, normal without limitations; 1, not completely normal but able to remain active and carry out fairly normal activities; 2, lacking energy for most activities but spending less than half the day in bed or sitting; 3, able to perform only small activities and spending most of the day in bed or sitting; and 4, bedridden. Source: Own elaboration.

In the specific analysis of symptomatic items related to irinotecan administration (*n* = 13), no statistically significant differences were observed in: eating problems (*p* = 0.271), loss of appetite (*p* = 0.294), constipation (*p* = 1.000), oral sores (*p* = 0.194), taste alterations (*p* = 0.293), unpleasant smells (*p* = 1.000), pain (*p* = 0.537), diarrhea (*p* = 0.209), dry mouth (*p* = 0.551), and dysphagia (*p* = 0.136) (Figure 2, Figure 3, Figure 4 and Figure 5).

Values close to statistical significance were observed for nausea (*p* = 0.089), associated with an increase of one point in the PG-SGA score, and vomiting (*p* = 0.087), which increased the score by up to three points (Figure 2, Figure 3, Figure 4 and Figure 5).

On the other hand, statistical significance was reached for early satiety (*p* = 0.041), which added one point to the scale score, and for progressive deterioration of functional capacity (*p* = 0.039), an item that may negatively increase the score by up to three points (Figure 2, Figure 3, Figure 4 and Figure 5).

When specifically comparing FOLFOX–irinotecan (*n* = 10) with FOLFOX alone (*n* = 26), a higher frequency of functional deterioration was observed in the combination group, contributing to higher overall PG-SGA scores.

### 3.4. Global Integration of Nutritional Scores

The combination of symptoms approaching significance (nausea and vomiting) together with the significant associations observed for early satiety and functional deterioration resulted in higher total PG-SGA scores in patients treated with irinotecan, particularly in combination with FOLFOX, compared with FOLFOX alone and XELOX. These findings were evident in the cohort assessed through both bioelectrical impedance analysis and PG-SGA, allowing characterization of a differential nutritional profile according to the administered therapeutic regimen.

## 4. Discussion

### 4.1. Prevalence and Prognostic Relevance of Malnutrition in Colorectal Cancer

In the analyzed cohort, a higher overall nutritional deterioration burden was observed in patients exposed to irinotecan, particularly those treated with the FOLFOX–irinotecan combination, where the increase in total PG-SGA scores was mainly driven by functional decline and the emergence of gastrointestinal symptoms impacting nutrition, such as early satiety, nausea, and vomiting. These findings may suggest that functional capacity may be an important contributor to nutritional deterioration in this therapeutic context, beyond isolated weight loss, reinforcing the concept of cancer-related malnutrition as a multidimensional process.

The high nutritional vulnerability observed in patients treated with irinotecan aligns with available literature in advanced colorectal cancer, where malnutrition prevalence ranges from 40% to 70%, depending on the diagnostic method used and the line of systemic treatment. Multicenter studies using PG-SGA have shown that high scores are independently associated with poorer treatment tolerance, dose reductions, and lower overall survival, even after adjusting for tumor stage and performance status [15,16,17]. Our results suggest a potential nutritional profile associated with irinotecan use, although this observation requires confirmation in larger cohorts.

From a prognostic standpoint, functional deterioration observed in patients treated with irinotecan has notable clinical relevance, as loss of functionality has been established as one of the most robust predictors of mortality and toxicity in gastrointestinal oncology, with a greater impact than BMI or isolated weight loss [18,19]. The relationship between functional status, muscle mass, and survival has been widely described in L3-level CT studies in metastatic colorectal cancer treated with fluoropyrimidine- and irinotecan-based chemotherapy [20,21].

### 4.2. Pathophysiology and Underlying Mechanisms

The nutritional deterioration profile observed in patients treated with irinotecan can be explained by a complex interaction between gastrointestinal toxicity, systemic inflammation, and accelerated muscle mass loss. Unlike oxaliplatin-only regimens, irinotecan induces more prolonged and clinically debilitating digestive toxicity, mediated by intestinal mucosal damage, activation of inflammatory pathways, and microbiota alterations, promoting anorexia, malabsorption, and early satiety [22,23].

The active metabolite SN-38 causes intestinal epithelial injury and release of proinflammatory cytokines, generating a hypercatabolic state that accelerates muscle proteolysis and reduces functional capacity. This process is amplified in advanced treatment lines, where cumulative toxicity from previous therapies diminishes patient physiological reserve, explaining the greater impact observed in the FOLFOX–irinotecan subgroup of our cohort. Pharmacokinetic studies have also shown that lean body mass is a key determinant of systemic exposure to irinotecan and its active metabolite, so patients with lower muscle mass experience higher toxicity for the same body surface area dose [24,25,26].

### 4.3. Influence of Age and Comorbidities

The mean age of the cohort, close to 65 years, situates the studied population in a clinical scenario where functional reserve and body composition directly affect treatment tolerance. Although no significant age differences between sexes were observed in our analysis, the literature shows that older patients have a higher risk of functional decline during irinotecan treatment, even in the absence of significant hematologic toxicity differences [27,28].

The coexistence of sarcopenia and frailty in metastatic colorectal cancer patients is associated with higher incidence of dose-limiting toxicity and early treatment interruptions, especially in irinotecan-containing regimens [20,29]. In this context, functional capacity assessed by PG-SGA could act as an integrative clinical marker of biological age, metabolic reserve, and therapeutic tolerance.

### 4.4. Nutritional Screening and Diagnostic Tools

Use of the PG-SGA enabled the identification of a nutritional deterioration pattern that would not have been detected by isolated anthropometric parameters. The relevance of the functional component was particularly evident in patients treated with irinotecan, where it was the main driver of the global score increase.

This finding aligns with international studies showing the superior predictive capacity of PG-SGA compared with simplified screening tools in actively treated oncology patients [15,30]. The inclusion of multifrequency bioelectrical impedance analysis in our cohort adds value by providing an objective measure of body composition, consistent with evidence indicating muscle mass as a primary determinant of chemotherapy toxicity and survival [20,31].

### 4.5. Clinical and Strategic Implications

The results have direct clinical implications for planning treatment in advanced colorectal cancer. Early identification of functional and nutritional deterioration in patients eligible for irinotecan could allow implementation of nutritional interventions and therapeutic exercise programs before dose reductions or treatment interruptions occur. Recent clinical trials have shown that multimodal interventions combining nutrition and exercise can improve chemotherapy tolerance and reduce toxicity in gastrointestinal cancer patients [31,32].

Our findings also support the need to systematically incorporate nutritional and body composition assessment into therapeutic decision-making, especially in the oxaliplatin–irinotecan sequence, where cumulative toxicity significantly affects functional capacity.

These findings should be interpreted within an exploratory framework, given the limited number of patients exposed to irinotecan and the absence of adjusted analyses.

### 4.6. Limitations

Although the sample size allows for consistent estimates aligned with previously published evidence, expanding the cohort in future studies would increase statistical power to detect smaller associations that may still be clinically relevant. The small number of patients exposed to irinotecan resulted in sparse data in several comparisons, which may limit the stability of statistical estimates. Although Fisher’s exact test was applied when appropriate, these analyses should be interpreted as exploratory. Similarly, the single-center design, while favoring standardization of procedures and consistency in diagnostic criteria and data collection, limits extrapolation to other care settings with different case mixes and organizational structures; multicenter studies would improve external validity and incorporate greater population heterogeneity.

The cross-sectional design provides an accurate snapshot of nutritional and functional status at a specific treatment time but prevents analysis of temporal trajectories and causal relationships between nutritional deterioration and clinical outcomes. Longitudinal studies with serial assessments would allow characterization of changes in body composition, functional capacity, and nutrition-impact symptoms across systemic treatment lines, particularly regarding cumulative irinotecan exposure.

Additionally, while this work focuses on clinical, functional, and validated nutritional assessment variables, the lack of detailed information on socioeconomic factors, lifestyle, habitual physical activity, and psychosocial factors may partially influence interpretation, given their recognized role in malnutrition risk, intervention adherence, and treatment tolerance. Future incorporation of these dimensions, together with systematic patient-reported outcomes and quality-of-life assessments using validated instruments, would allow a more comprehensive, patient-centered understanding of the clinical impact of nutritional deterioration.

Importantly, the cross-sectional design of the study precludes establishing temporal or causal relationships between irinotecan exposure and nutritional or functional deterioration. Additionally, residual confounding cannot be excluded, as patients receiving irinotecan may represent a more heavily pretreated or clinically vulnerable subgroup.

Therefore, the observed associations should be interpreted as hypothesis-generating and require validation in prospective, adequately powered studies.

The absence of multivariable adjustment limits the ability to control for potential confounding factors.

## 5. Conclusions

In this exploratory cross-sectional study, exposure to irinotecan-containing regimens may be associated with a pattern of greater nutritional burden, primarily characterized by functional decline and selected gastrointestinal symptoms affecting food intake.

However, given the limited number of patients receiving irinotecan and the unadjusted nature of the analyses, these findings should be interpreted with caution and considered hypothesis-generating rather than confirmatory. Further prospective studies with larger sample sizes are required to confirm these observations.

## Data Availability

The original contributions presented in this study are included in the article. The datasets generated and/or analyzed during the current study are available from the corresponding author upon reasonable request.
